# A Systematic Review of the Experiences and Support Needs of Informal Caregivers for People Who Have Attempted Suicide or Experienced Suicidal Ideation

**DOI:** 10.3390/ijerph19095181

**Published:** 2022-04-24

**Authors:** George Lavers, Karl Andriessen, Karolina Krysinska

**Affiliations:** 1 Melbourne Medical School, The University of Melbourne, Parkville, VIC 3010, Australia; glavers@student.unimelb.edu.au; 2 Centre for Mental Health, Melbourne School of Population and Global Health, The University of Melbourne, Parkville, VIC 3010, Australia; karl.andriessen@unimelb.edu.au

**Keywords:** informal caregiver, suicidality, attempted suicide, suicidal ideation, lived experience, support needs

## Abstract

Informal caregivers include family, friends, and significant others who provide important support for people who have attempted suicide or experienced suicidal ideation. Despite the prevalence of suicidal behaviour worldwide, they remain an understudied population. This review aimed to synthesise the literature on the experiences and support needs of informal caregivers of people who have attempted suicide or experienced suicidal ideation. We conducted a systematic review according to PRISMA guidelines. Searches of peer-reviewed literature in Medline, Emcare, Embase, EBM Reviews, and PsycINFO identified 21 studies (4 quantitative and 17 qualitative), published between 1986 and 2021. Informal carers commonly reported symptoms of depression and anxiety, for which they receive little assistance. They also expressed a desire for more involvement and education in the professional care of suicidality. Together, the studies indicated a need to improve the way informal caregiving is managed in professional healthcare settings. This review identified potential avenues for future research, as well as broad areas which require attention in seeking to improve the care of suicidal people and their caregivers.

## 1. Introduction

More than 700,000 people die by suicide worldwide each year [1]. In Australia, suicide is the leading cause of death in people aged 15–44, though affects a higher proportion of the older population [2]. In 2020 the age-standardised suicide rate for Aboriginal and Torres Strait Islander people was 27.9 per 100,000 vs. 12.1 per 100,000 people for the total Australian population [2]. However, the phenomenon of suicidality comprises a wider range of behaviours including suicidal ideation and non-lethal attempts. In 2019–2020 there were over 28,600 hospitalisations for self-harm in Australia, which provides an estimate of the extent of the phenomenon [3]. Given the magnitude of suicidal behaviour in the population, there is a clear need for prevention and care of suicidality across diverse ages and backgrounds.

Informal caregivers in the context of suicidality are a diverse range of people including “family, friends and significant others who support a loved one after a suicide attempt” [4], p. 4 who play a key role in the care of individuals/people who have suicidal thoughts or engage in suicidal behaviour [4,5]. In their 2021 review, Simes and colleagues found that most suicidal youth, including in Australia, do not receive professional mental healthcare, and that family-centred therapy is an important and underutilised form of care [6]. Indeed, much of the care of suicidal people occurs in an informal setting [7]. Informal caregivers, including family members, are therefore of key importance in suicide intervention [8].

Despite this, studies tend to overlook informal caregivers as a population, not just in the context of suicide research and prevention, but across many other health contexts [9,10]. The literature on this topic has gradually been increasing, leading to consideration of the value of family-centred care in the context of suicidality and self-harm [5,6]. However, despite the development of the field, there has been no systematic review of literature focussed on informal caregivers’ experiences and support needs while caring for a person at risk of suicide. It has been noted in other contexts that the vast amount of daily care provided by informal caregivers without support can lead to significant mental health burden [11]. Therefore, the lack of widespread understanding of informal caregiving in the context of suicidality may hide a significant public health issue.

Caregiver stress has been acknowledged as a significant source of psychological and physical morbidity in caregivers of diverse groups such as cancer patients [12,13] and dementia patients [14]. The effects of caregiver stress have been characterised as “widespread and unnecessary suffering, isolation, fear, error, and at times bankruptcy” [15], p. 1021. This highlights the effect of caregiver stress on both the caregivers themselves, and those being cared for. 

This systematic review aims to synthesise the quantitative and qualitative research literature on the experiences and support needs of informal caregivers in the context of suicidality, and identify their common experience, support needs, and support received. This will be explored with the goal of formulating implications for practice and research.

## 2. Materials and Methods

### 2.1. Search Strategy

The review adhered to the PRISMA guidelines [16] and was registered in the PROSPERO database (CRD42021274108). The review involved systematic searches of the literature in Medline, Emcare, Embase, EBM Reviews and PsycINFO (all accessed via OVID in August 2021). The search in Medline used MeSH and text words: (attempted suicide.mp. OR Suicide, Attempted/OR suicide attempt.mp. OR non-fatal suicidal behaviour.mp. OR Suicidal Ideation/OR Suicidal behaviour.mp. OR self-harm.mp. OR self-injury.mp. or Self-Injurious Behavior/) AND (Family/or family.mp. OR informal carer.mp. OR carer.mp. OR caregiver*.mp. or Caregivers/OR spouse*.mp. OR Spouses/OR parent.mp. OR Parents/OR sibling*.mp. or Siblings/OR Grandparents/or grandparent*.mp. OR partner.mp. OR lived experience.mp) AND (support needs.mp. OR needs.mp.). A similar search string including headings and keywords was used in the other databases. The searches were limited to peer-reviewed publications in English but not by date of publication. 

**Figure 1:**
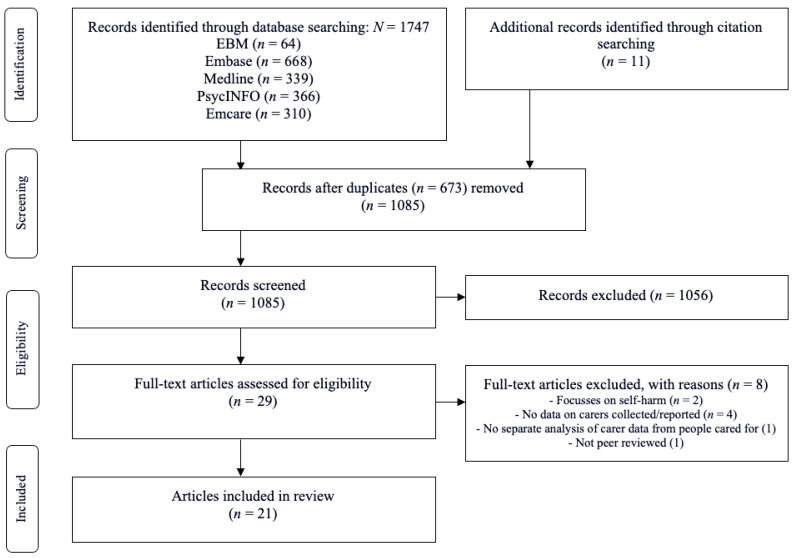
PRISMA flow diagram.

One researcher (G.L.) conducted the searches, and screened titles and abstracts of the leads regarding their potential eligibility. Researchers G.L. and K.K. assessed full texts of the selected abstracts against the inclusion and exclusion criteria. Any disagreement was resolved through discussion with the third researcher (K.A.). Researcher G.L. hand searched the references of the included studies and review papers. Figure 1 details the search and selection process. 

### 2.2. Inclusion and Exclusion Criteria

Studies were included if: (i) the study population consisted of informal caregivers of people who have attempted suicide and/or experienced suicidal ideation, (ii) the study used quantitative, qualitative or mixed methods, (iii) the study provided empirical data on the support needs of the study population, (iv) the study was published in English, (v) the study was peer-reviewed. 

Studies were excluded if the study used other methods such as case studies or literature review.

### 2.3. Data Extraction

Researchers G.L. and K.K. independently extracted the following data by listing and comparing the data and main findings with each re-reading of the corpus: author, year and location of study, sample size, participants’ sex, age, caregivers’ relationships, study design, main results, and study limitations. Any disagreement was resolved through discussion with the third researcher (K.A.).

### 2.4. Quality Assessment

Two researchers (G.L., K.K.) independently conducted the quality assessment and resolved disagreements through discussion with the third researcher (K.A.). No eligible study was excluded based on its quality. The Newcastle-Ottawa Quality Assessment Form for Cohort Studies [17] was used to assess quantitative studies. The scale comprises eight items across three domains: (1) selection (four items), (2) comparability (one item), and (3) outcome (three items). Scores in each domain were summed to determine study quality as good, fair, or poor. The interrater reliability was substantial (κ = 0.77).

The qualitative studies were assessed using the Consolidated Criteria for Reporting Qualitative Research (COREQ) [18]. The instrument consists of thirty-two items across three domains: (1) research team and reflexivity (eight items), (2) study design (fifteen items), and (3) analysis and findings (nine items). For each study, we calculated the number and percentage of items satisfied within each domain and across all domains. The interrater agreement was high (κ = 0.85).

## 3. Results

### 3.1. Study Characteristics

This review identified 21 studies that investigated the experiences and support needs of informal caregivers of people experiencing suicidality. Four studies included quantitative data [19,20,21,22]. The other 17 studies were qualitative [23,24,25,26,27,28,29,30,31,32,33,34,35,36,37,38,39]. In general, qualitative studies involved interviews or surveys with thematic analysis (see Table 1).

The studies found were largely conducted in Western nations, including five studies in the USA [19,24,25,26,27], four in Australia [23,28,29,30], four in Sweden [20,21,22,31], one in the UK [32], two in Northern Ireland [33,34], two in Denmark [35,36] and one each in Canada [37], South Africa [38] and Taiwan [39].

A wide variety of people act as informal caregivers for suicidal people, though caregivers were predominantly female in all studies except the study by Sun and colleagues, in which nine participants were men and six were women (39). Two studies recruited only female caregivers [37,38] while the rest included mostly female caregivers, often approaching nearly 100%. For example, in Wayland et al.’s Australian survey with over 700 respondents, 86.9% were female [30].

Most studies chose to investigate parental caregivers of suicidal youths or adult children. Among two of the studies which reported recruiting a variety of caregiver relationships, caregivers were most often parents (44% [22] and 37.6% [19] of participants), while spouses predominated in the others [21,31,39]. Studies also included wider family members and friends [20,21,22,25,26,27,31,33,39]. Wayland’s study was unique in that the interviewees were mostly children providing care for parents experiencing suicidality [30].

### 3.2. Quality Assessment

Appendix A (Table A1) presents the methodological quality of the four quantitative studies. One study received a rating of ‘good’ quality [19], and the others rated as ‘poor’ [20,21,22]. Studies tended to score well in the ‘selection’ domain, but tended to score poorly in the ‘comparability’ and the ‘outcome’ domain by relying on self-reported outcomes. Appendix B (Table A2) outlines the quality assessment of the 17 qualitative studies. The studies reported between 44% [27] and 94% of the COREQ criteria [28]. Most studies reported only few items across the ‘research team and reflexivity’ domain (on average 38% of items were reported). However, studies reported more items in the ‘study design’ (60%) and ‘analysis and findings’ (75%) domains.

### 3.3. Study Findings Quantitative Studies

#### 3.3.1. Emotional Burden

Psychological and practical supports were a noted requirement of caregivers [21]. Magne-Ingvar and colleagues found that 55% of caregivers had provided psychological support and one in three had helped with practical matters [21]. Of these people, 57% described it as a burden. Accordingly, themes of stress, and anxiety were noted as central elements of the informal caregiver experience, and these psychological stresses were significant enough to manifest at times in physical ill health [21,22].

Regarding the source of this stress, disruption to family life was reported among the quantitative studies [21]. In addition to stress and anxiety, themes of depression and low mood were noted caregiver emotional responses in two studies [19,21]. Notably, Chessick and colleagues identified that these responses are modulated by factors specific to the suicidal person: caregivers tended to experience higher levels of depression when caring for people with worse daily function due to their mental status, or less educational attainment [19]. 

An additional reported source of stress is that of reduced work and leisure time for caregivers: 28% of caregiver relatives in Kjellin and Östman’s study had reduced leisure time, with a smaller amount reporting reduced time at work and one third being unable to spend time alone [20].

#### 3.3.2. Desired Supports

Caregivers requested a need for personal care and support, with Kjellin and Östman noting that their results “corroborate the need for psychiatric services to involve and support relatives of psychiatric patients with suicidal behaviour.” [20], p.11. In one study by Magne-Ingvar and colleagues, 53% of caregivers desired counselling together with the suicidal person, with a smaller proportion expressing a desire for more private counselling [21]. This study again noted caregivers’ wishes to be more involved in the professional care of their suicidal loved ones [21].

There is also a possibility that caregiver strain would be relieved by provision of professional care to those experiencing suicidality. In addition to their personal needs, Magne-Ingvar and colleagues sought for suggestions by caregivers of future supports that would benefit significant others experiencing suicidality [21]. Notably, most participants in this study had significant others already receiving such treatment. 

### 3.4. Study Findings Qualitative Studies

#### 3.4.1. Emotional Burden

The studies noted several caregiver responsibilities which had the potential to contribute to caregiver emotional burden. These included managing the risk of suicide in care recipients as well as psychological and practical supports [30]. Wayland and colleagues [30] enumerated specific domains which caregivers had to take on in caring for family members; these included financial assistance/decisions, transport, phone calls and life advice. 

Accordingly, stress, fear, anxiety, and hypervigilance were central elements of the informal caregiver experience in most studies [24,26,28,29,30,33,34,36,38,39]. Chronic stress, at times manifesting in post-traumatic stress disorder, was ubiquitous in one study [29]. In addition, Sun and colleagues attributed a certain amount of stress to the open family environment particular to the prevailing culture in Taiwan, where that study was carried out [39]. This family environment was viewed as conflicting with parents’ desires to remain on guard for their suicidal family members [39]. Meanwhile, Fogarty and colleagues described tensions as arising mainly from the difficulty in managing suicide risk while maintaining a working relationship with the care recipient [23]. 

The stress experienced by caregivers can be further characterised as a “double trauma”, with the added damage that caregiving inflicts on the family and relationships [36]. Daly’s study of maternal caregivers defined themes of “failure as a good mother” and “rejection” by their children [37]. A disrupted family life was corroborated by various studies [28,29,32,34,38]. Importantly, relationship disruption was not necessarily a ubiquitous experience. Roach identified that youth peer caregivers often felt “honoured” to provide support and that the experience was overall positive for their relationships [26].

#### 3.4.2. Desired Supports

Caregivers commonly requested a need for emotional support, including from dedicated psychiatric services [24]. McLaughlin and colleagues described simple measures to reduce burden such as follow-up calls from healthcare staff to more isolated caregivers, as well as more complex supports such as respite services [33]. 

There are more basic measures that improve caregiver perceptions of the healthcare system. Inscoe and colleagues identified that caregivers positively regarded clinician empathy, validation and nonjudgment [24], while others reported that simply being asked if they were coping at home would have been worthwhile [30,33]. McLaughlin and colleagues seem to have found a lack of support to be a ubiquitous and damaging experience among participants, with one participant stating “THERE WAS NOBODY TO TURN TO” [participants’ emphasis] ([33], p. 214).

Many studies identified caregivers’ wishes to be more involved in the professional care of their suicidal loved ones [24,28,33]. In one study, 37.6% of family members of suicidal individuals felt emergency department staff did not wish to communicate with them about their loved one [27]. McLaughlin and colleagues noted this type of involvement in care is possible for suicidal children, but that automatic involvement of caregivers may abruptly stop when children reach adulthood [33]. They also found that more than one in five participants did not feel they had been well treated by staff [33]. Other studies noted further departures from expected care. Giffin and colleagues reported that hospital admissions often did not meet the needs of the family, being only brief or in response to crisis [29], while Dempsey and colleagues found that clinicians needed to better explain the reasons for continued admission vs. discharge [28]. 

Discharge from hospital services following a suicide attempt may be a significant trigger for caregiver distress, in part since assessment of suicide risk in a hospital environment did not necessarily equate to that of the home environment [30]. Many studies identified that discharge often occurred without informal caregivers receiving effective education about managing suicide risk out of hospital [28,29,32,33,38]. Others highlighted the importance of education about suicidality “warning signs” [24,26]. In the study by Roach and colleagues, youth peer caregivers were cognisant of a need to involve adults when suicidality became active [26]. 

Caregivers often experienced professional care for suicidal patients as fragmented and un-cooperative, at times leading to contradictory health advice [28,29,33]. Caregivers have reported difficulties in navigating a complicated mental health system [23,24], and feeling that clinicians do not know what constitutes safe practice for informal caregivers [28]. This dependence on community services which may fail to provide sufficient care for suicidal people was noted as a key issue by Fogarty and colleagues [23]. Study participants therefore expressed a desire for skills training for informal caregivers to reduce reliance on services and improve collaboration with the services [23,30]. 

## 4. Discussion

This review identified 21 studies published within the last four decades, across nine highly developed nations. The caregivers studied differed in age, nationality, relationship, and so on. However, the preponderance of female informal caregivers was similar across studies. Some studies noted the relative lack of male caregivers as a limitation [24,28] and Inscoe and colleagues noted that “more research is needed to understand the role of male caregivers in accessing and participating in treatment” ([24], p. 6.) However, there is a similar preponderance of female caregivers in the context of other medical conditions or disabilities, such as elderly people suffering from dementia or other physical conditions, and this possibly reflects traditional societal gender roles [40]. 

The reviewed studies indicated that the psychological impact of suicidality on informal caregivers should not be underestimated. Suicidality affects a wide range of people surrounding the suicidal person, including people with pre-existing mental health issues [31]. Caregiving stress was reported to disrupt family dynamics as well, supporting previous studies that stigma around suicide may impact families as frequently as the suicidal person [41]. This disruptive stress is an impediment to the care of suicidal people, and potentially a real danger for caregivers; several studies discuss the possibility of burnout and resentment, or indeed the possibility of passive “death wishes” developing in unsupported caregivers [25,31,39].

Regarding depression and low mood, Chessick and colleagues identified that these emotional responses are modulated by factors specific to the suicidal person [19]. Caregivers tended to experience higher levels of depression when caring for people with worse daily function due to their mental status, or less educational attainment [19]. However, these mood states do not necessarily arise *de novo* when people take on a caregiving role. Wolk-Wasserman observed that a majority of family/spousal caregivers experienced their own psychiatric issues such as substance use problems, psychosis, and previous suicide attempts [31].

In addition to social context [39] and suicide risk [23], this review identifies gender inequity as a potential contributor to caregiver stress. Chessick and colleagues suggested that male spousal caregivers might “assume that traditionally female role functions are relatively easily performed” possibly leading to greater disappointment and caregiver burden when that expectation is not met by the female partner ([19], p. 489). Buus and colleagues noted that between parental caregivers, the stereotype of women needing to talk about issues more than men created relationship conflict, which then contributed to caregiver stress [36]. The gender-related element of suicidality is further reinforced by the fact that women tend to be rated as more frequently suicidal by certain measures than men [42]. Less reliance on traditional gender roles might reduce caregiver burden by spreading care and stress more equitably within families.

The significant psychological burden that suicidality has on informal caregivers suggests a need for professional interventions for caregivers. McLaughlin and colleagues indicated that perceived stigma often prevented family caregivers from seeking help from healthcare services, and that clinician warmth and empathy are important in encouraging help seeking [34]. It is therefore recommended that clinicians enquire about caregiver coping to create a permissive help-seeking environment. 

The need for professional interventions is supported by Wolk-Wasserman’s finding that most caregivers for suicidal people themselves had psychiatric issues [31]. Indeed, it is possible that targeting psychosocial interventions to family caregivers may be a worthwhile public health intervention given the indications of a partial heredity of suicidality [43], and the likelihood of shared socio-economic status between caregivers and care recipients in the studies examined. As such, healthcare workers should be prepared to identify caregivers suffering unmanageable stress and to direct them to relevant services [44]. Screening procedures for caregiver mental health are valuable and clinicians may benefit from education on assessment and intervention strategies as has been described in other caregiving contexts [44]. 

Informal caregivers who choose to seek help should have access to formal support services. These could take the form of respite care [34] or psychological counselling [21,45]. However, in certain locations, the prohibitive cost of psychological support is a further barrier to formal care [24]. As a result, the provision of informal caregiver support services must be facilitated by systemic support such as financially equitable healthcare or financial aid, as has been identified in palliative caregiving contexts [46]. 

The array of mental health services is confusing to many informal caregivers [23,24] and efforts should be made to reduce the impact of this issue. Clear, accessible information, for example on a dedicated government website, about what options are available and appropriate at each step of a suicidal person’s healthcare could alleviate the caregivers’ stress and improve care delivery. However, some studies have reported caregivers’ perception that care services are disparate and fragmented, creating a barrier to access [28,29,33]. This is a systemic health issue which has also been found in other caregiving contexts such as chronic disease [47,48], suggesting the need for systemic healthcare changes and streamlining.

Informal caregivers often express a wish to be included in the formal care [21,24,28,33]. However, involvement of informal caregivers in formal settings is not the default when the suicidal person is an adult without legal guardians [33]. Indeed, in some contexts even caregivers of children experience this lack of involvement [49]. This issue can in part be addressed if healthcare professionals offer warmth and empathy to informal caregivers, thereby creating an environment where informal caregivers feel as though their unique perspective is heard, and their distress is acknowledged [49]. 

This review found that informal caregivers often feel ill-prepared for the post-discharge period [26,27,30,33], particularly around identifying active suicidality [24,26]. Indeed, it has been noted that the efficacy of suicide risk assessment in the clinical setting is limited [50]. This highlights a clear area for reducing informal caregiver burden; healthcare providers should offer basic education about assessing risk and thereby improve the safety of their clients after discharge from acute care. Clear guidelines for formal caregivers are required to ensure safety and accuracy of this education, and the need for such guidelines is further highlighted by the finding that formal caregivers may differ widely in their advice for informal caregivers [28]. 

### Limitations

This review was limited to English language literature. Future reviews could involve additional databases or studies in other languages. Most studies were qualitative, or cross-sectional, and involved mostly female participants from western countries. The interview methodologies of the quantitative studies are variable and lack comparability and are therefore not conclusive. Individual studies possessed their own inherent limitations, including sample size or method, which are outlined in Appendix A and Appendix B. Quality assessment of all studies was performed in lieu of risk of bias analysis. In the Australian context, studies were predominantly of Caucasian subjects and therefore do not capture the range of cultural diversity present in the wider Australian population. Overall, no study focussed on First Nations populations despite the known high prevalence of suicide in this group.

## 5. Conclusions

The extent of psychosocial stress endured by informal caregivers of people experiencing suicidality is significant. Despite this, they remain a poorly studied population. More research is needed, including studies of caregiver experiences and intervention studies aimed at determining how best to meet their needs.

The identified needs and wishes of informal caregivers suggest some actions that can be taken by healthcare professionals involved in the care of suicidal people. Healthcare professionals should be prepared to screen these caregivers for issues including depression and anxiety, with offers of formal assistance where necessary. Informal caregivers routinely express a desire for involvement in formal care, so efforts should be made where possible to seek insight and assistance from willing informal caregivers. Healthcare professionals should be able to provide education about managing suicide risks and active suicidality when asked.

Finally, the widespread lack of understanding of these informal caregivers suggests a potentially untapped wealth of experience. Informal caregivers in the setting of suicidality have described significant benefits of peer support e.g., [32], and this represents a potential avenue for improving quality of life for this population.

## Figures and Tables

**Table 1:** Summary of included studies.

Author (s), Year, Location	Sample Size	Demographics	Caregiver Relationship (S)	Study Design	Main Results
Quantitative Studies					
Chessick et al. (2007) USA [19]	*n* = 500	345 females (remaining genders not reported) Mean age = 50.2 ± 13.1 years 439 Caucasians	188 parents 182 spouses 28 siblings 22 children 80 others	SBAS (Social Behaviour Assessment Schedule) assessed caregiver burden over three domains CES-D (Centre for Epidemiological Studies of Depression Scale) General Health Scale from the Medical Outcomes Study (MOS) 36-iten Short-Form Health Survey Cross-sectional analysis	Caregivers of younger people and/or people with lower GAF scores were more likely to report increased burden (*p* < 0.05). Caregivers of people with lower GAF scores and/or less education were more likely to report higher levels of depression (*p* < 0.05).
Kjellin and Östman (2005) Sweden [20]	*n* = 155 (N.B. includes patients *and* relatives)	51% females 49% males Age group mode = 40–59 years Ethnicity not reported	29% spouses 27% siblings or other relatives 12% sons or daughters 5% close friends Remaining percentage unreported	Semi-structured questionnaire Cross-sectional analysis	Relatives of people with suicide attempts more often than other relatives stated they had been prevented from having own company (52% vs. 29%) More often worried about suicide attempts (59% vs. 25%) More often had mental health problems of their own (56% vs. 35%)
Magne-Invar et al. (1999) Sweden [21]	*n* = 84	Not reported	37 parents 23 partners 24 others (10 adult children, 2 siblings, 5 ex-partners and 7 friends)	Semi-structured interviews Cross-sectional analysis	77% worried the person was going to hurt themselves again. 25% stated they had frequently been worried. Most had someone to turn to themselves, but 46% would have liked professional counselling (more often partners) with the suicidal person, shortly after the attempt. 45% considered treatment of the suicidal person to be insufficient. 22% felt they themselves hadn’t been well treated by staff, 2/3 not enough information. 42% desired involvement in outpatient treatment, only 11% were involved. 17% had “not good” general well-being, but 2/3 had mental symptoms.
Magne-Ingvar and Öjehagen (1999) Sweden [22]	*n* = 81	49 females 32 males Ages not reported Ethnicity not reported	Partners/ex-partners = 31 Parents = 30 Grown-up children = 12 Siblings = 2 Friends = 6	Semi-structured interviews and questionnaire Cross-sectional analysis	55% had provided the suicidal person with psychological support. 1/3 had helped with practical matters. 75% stated they were upset, worried or shocked following the suicide attempt. 16% reported they felt physically unwell and about 25% of them reported sleeping, mood and/or appetite problems. 41% had other personal problems, mostly relationship or vocational problems. 57% who had given support (36% of all caregivers) stated that this was burdensome. Most stated that it was helpful to talk to a professional soon after the suicide attempt. 53% wanted counselling together with the suicidal person. 37% wanted individual counselling. 6% were uncertain if they needed more professional support.
**Qualitative studies**					
Byrne et al. (2008), Ireland [32]	*n* = 15	Not reported	Parents	Focus group meeting Transcript-based conceptual analysis	Support groups should address: Need for/Lack of support from services. Benefits of peer support. Emotions including: guilt, isolation, fear, frustration, lack of confidence. Disruption to whole family. Psycho-education for managing self-harm episodes. Other: beliefs about self-harm, school (lack of support).
Buus et al. (2013) Denmark [36]	*n* = 14	9 females 5 males Ages not reported Ethnicity not reported	Parents	Focus group interviews Thematic analysis	Emotional responses and stress. Double trauma: effects on families and relationships.
Cerel et al. (2006) USA [27]	*n* = 254 carers	213 females 41 males 63% >45 years old 94% white, non-Hispanic	Family members and friends	Online survey Statistical analysis and categorisation with iterative process	37.6% reported ED staff did not want to communicate with them about their loved one. Most positive comments concerned positive experiences with staff Family members’ most frequent negative comments concerned a perception of unprofessional staff behaviour.
Daly (2005) Canada [37]	*n* = 6	6 females Ages = 32–45 years Ethnicity not reported	Mothers	Unstructured interviews Thematic analysis	Failure as a good mother. “The ultimate rejection”. Feeling alone in the struggle. Helplessness and powerlessness in the struggle. Cautious parenting. Keeping an emotional distance.
Dempsey et al. (2019) Australia [28]	*n* = 8	7 females 1 male Mean age = 52.5 years Ethnicity not reported	Parents	Semi-structured interviews Thematic analysis	Confusion about contact numbers. Preference for printed or online information varied. Caregivers’ information needs were discordant with clinicians’ expectations, caregivers wanted more info about expectations of treatment. Reassurance and emotional support. How to get help. Two-way sharing of information, feedback. Discharge needs. General caregiver challenges (including own emotions, family and life, suicide risk management). Anxiety, guilt, shame, isolation, bewilderment. Struggled with balancing time. Gaps in suicide management competence, removing dangers.
Fogarty et al. (2017), Australia [20]	*n* = 47	26 females (remaining genders not reported) Median age = 47 years Ethnicity not reported	Family and friends	Patient Health Questionnaire 9, GAD-7 Semi-structured interview and focus groups Qualitative secondary analysis	5 processes caused tension: Respect for privacy vs. vigilance in risk monitoring. Differentiating normal vs. risky behavioural change. Familiarity vs. anonymity in risk disclosure. Respecting autonomy vs. imposing constraints to limit risk. Dependence on vs. perceived failures of community services.
Giffin, J (2008), Australia [29]	*n* = 4	3 females 1 male Ages not reported Ethnicity not reported	Parents	Unstructured, in-depth interviews Grounded theory	Chronic stress and intrusive thoughts were ubiquitous. Emotional strain and exhaustion—one parent diagnosed with PTSD. Treatments that discourage psychiatric admission or include only crisis admissions were not helpful and burdened family. Health professionals gave conflicting messages about what benefits suicidal person. Caring for child created strain on couple relationships. Different views from family members created tension. Inconsistency from service providers and lack of sensitivity for carer needs after discharge.
Inscoe et al. (2021) USA [24]	*n* = 13	12 females 1 male 39–57 years White (*n* = 6), Black or African American (*n* = 4), American Indian or Alaska Native (*n* = 2), Asian (*n* = 1)	Not stated	Semi-structured interviews Iterative data analysis.	Importance of caregiver involvement in trauma-informed care. Need for emotional support to better care for their children. Instrumental support needed: education about suicide and trauma. Important clinician behaviours: nonjudgment, empathy, validation + understanding of traumatic stress impacts. Barriers: difficulties to navigate the mental health system; costs.
McLaughlin et al. (2014) Northern Ireland [34]	*n* = 18	14 females 4 males Ages = 25–78 years Ethnicity not reported	Not stated	Semi-structured interviews Thematic analysis	Family burden. Competing pressures. Secrecy and shame. Helplessness and guilt.
McLaughlin et al. (2016) UK [33]	*n* = 18	Genders not reported Ages = 25–78 years Ethnicity not reported	Siblings, partners, parents, children, etc.	Semi-structured interviews Thematic analysis	Having practical support, respite, advice. Feeling acknowledged and included: “overwhelming desire to be involved in the hospitalised care” but were suddenly excluded after family member turned 18. Needing support themselves. Healthcare staff could work together better and have better internal continuity of care.
Ngwane et al. (2019) South Africa [38]	*n* = 10	10 females Ages = 29–59 years 3 from Tsonga culture group and 7 from Tswana	10 parents	Semi-structured interviews Thematic analysis	Post-traumatic experiences. Regret, self-blame, and guilt. Fear anxiety. Disturbed family relationships. Coping mechanisms (including need for counselling).
Nosek (2008) USA [25]	*n* = 17	Not reported	7 spouses 5 parent(s) 1 sibling 1 adult daughter	Semi-structured interviews Grounded theory analysis	Initial “*not knowing*” progresses to gaining awareness of their loved one’s suicidality. This leads into a cyclical process of “*taking action*” and gaining more awareness, which can involve simply “*knowing*” about the issue, or attainment of a deeper level of “*understanding*” about the issue. These processes involved constant watching/waiting to reassess approaches. This led to eventual burnout and “*reaching limit*”. This could at times progress to “*not wanting to know*” about the issue at all.
Nygaard (2019) Denmark [35]	*n* = 19	12 females 7 males 42–81 years old Ethnicity not reported	Parents	Semi-structured interviews Thematic analysis	A sense of solidarity between partners; challenges developed when the partners did not receive basic communication and acknowledgement. Responding to different reactions and coping strategies.
Roach et al. (2020), USA [26]	*n* = 5	3 females 2 males 16–17 years old Ethnicity not reported	5 school friends (“kids helping kids”)	Unstructured interviews Phenomenological research design	Being fearful. Maintaining vigilance. Seeking knowledge. Keeping secrets. Involving others. Setting boundaries. Feeling honoured.
Sun et al. (2008) Taiwan [39]	*n* = 15	9 males 6 females Mean age = 50 years Ethnicity not reported	6 partners 4 parents 3 siblings 2 children > 20 years	Interviews Grounded theory	Family environment is “open” s could not monitor whereabouts and behaviours). Chinese culture stigmatises suicide. Effects of and barriers to caring (include support systems and coping strategies). On guard day and night to ensure suicidal relatives felt safe. Maintaining activities of daily living promoted physical recovery. Impending burnout; family members were both physically and emotionally exhausted.
Wayland et al. (2020) Australia [30]	*n* = 758 online survey participants + 32 interviewees	659 females 81 males 13 “other” 5 transgender individuals Ages not reported 2 Aboriginal or Torres Strait Islander individuals	190 children 176 friends 85 partners 71 parents 51 other family members 45 siblings	Cross-sectional online community survey and semi-structured interviews Thematic analysis	Needing to take on a practical caring role (financial, transport, phone calls, booking appointments, day-to-day life advice, healthcare cost decisions, similar roles to case managers). Lack of agreed role (meant lack of requisite skills). Hypervigilance. Balancing personal safety vs. independence. No safety net for carers. Post-discharge as a touchpoint for carer distress. What helped/didn’t help (wanted to be asked if they were well equipped to manage “suicide watch”, required individualised support away from the person they were supporting, managing privacy and confidentiality to better involve carer).
Wolk-Wasserman (1986) Sweden [31]	*n* = 70	Not stated	24 partners 23 parents 23 other relatives (sibling, adult child, sister-/brother-in-law) or friends	Semi-structured interviews Descriptive qualitative analysis	Protracted indirect verbal communication. Absence of dialogue. Ambivalence and aggressiveness. Development of reactions to suicidal communications: (i) silence and increased tension, (ii) obvious ambivalence, (iii) visible aggressiveness in some cases. Many partners had psychiatric issues of their own in need of treatment.

## Appendix A

**Table A1:** Quality assessment ^1^ of quantitative studies.

Topic	Chessick et al., 2007 [19]	Kjellin & Ostman, 2005 [20]	Magne-Ingvar & Ojehagen, 1999 [21]	Magne-Ingvar & Ojehagen, 1999 [22]
Selection				
1. Representativeness of the exposed cohort				
a. Truly representative (one star)				
b. Somewhat representative (one star)	X	X	X	X
c. Selected group				
d. No description				
2. Selection of the non-exposed cohort				
a. Drawn from the same community as the exposed cohort (one star)	X	X		
b. Drawn from a different source				
c. No description			n/a	n/a
3. Ascertainment of exposure				
a. Secure record (e.g., surgical record) (one star)	X	X		
b. Structured interview (one star)			X	X
c. Written self-report				
d. No description				
e. Other				
4. Demonstration that outcome of interest was not present at start of study				
a. Yes (one star)				
b. No	n/a	n/a	n/a	n/a
Comparability				
1. Comparability of cohorts on the basis of the design or analysis controlled for confounders				
a. The study controls for age, sex and marital status (one star)	X			
b. Study controls for other factors (list) (one star)	X			
c. Controls are not comparable		X	n/a	n/a
Outcome				
1. Assessment of outcome				
a. Independent blind assessment (one star)				
b. Record linkage (one star)				
c. Self-report		X	X	X
d. No description				
e. Other	X			
2. Was follow-up long enough for outcomes to occur				
a. Yes (one star)	X	X	X	X
b. No				
Indicate the mean duration of follow-up and a brief rationale for the assessment above	Life-time prevalence	<1 month; >1 month	Within days	One year
3. Adequacy of follow-up of cohorts				
a. Complete follow-up, all subjects accounted for (one star)	X		X	
b. Subjects lost to follow-up unlikely to introduce bias, number lost less than or equal to 20% or description of those lost suggested no different from those followed (one star)		X		X
c. Follow-up rate less than 80% and no description of those lost				
d. No statement				
Stars				
Selection	3	3	2	2
Comparability	2	0	0	0
Outcome	2	2	2	2
Rating	Good	Poor	Poor	Poor

^1^ Newcastle-Ottawa Quality Assessment Form for Cohort Studies [17]. Note: A study can be given a maximum of one star for each numbered item within the Selection and Outcome categories. A maximum of two stars can be given for Comparability. Thresholds for converting the Newcastle-Ottawa scales to AHRQ standards (good, fair, and poor): Good quality: 3 or 4 stars in selection domain AND 1 or 2 stars in comparability domain AND 2 or 3 stars in outcome/exposure domain. Fair quality: 2 stars in selection domain AND 1 or 2 stars in comparability domain AND 2 or 3 stars in outcome/exposure domain. Poor quality: 0 or 1 star in selection domain OR 0 stars in comparability domain OR 0 or 1 stars in outcome/exposure domain.

## Appendix B

**Table A2:** Quality assessment ^1^ of qualitative studies.

	Topic	Buus et al., 2014 [36]	Byrne et al., 2008 [32]	Cerel et al., 2006 [27]	Daly, 2005 [37]	Dempsey et al., 2019 [28]	Fogarty et al., 2018 [23]	Giffin, 2008 [29]	Inscoe et al., 2021 [24]	McLaughlin et al., 2014 [34]	McLaughlin et al., 2016 [33]	Ngwane et al., 2019 [38]	Nosek, 2008 [25]	Nygaard et al., 2019 [35]	Roach et al., 2020 [26]	Sun et al., 2008 [39]	Wayland et al., 2020 [30]	Wolk-Wasserman, 1986 [31]
	Domain 1: Research team and reflexivity																	
	*Personal characteristics*																	
1	Interviewer/facilitator	p.^2^ 825	p. 496		p. 28	S.1	p. 263	p. 134	p. 2			p. 376	p. 38	p. 135		p. 1947	p. 663	p. 484
2	Credentials	p. 823	p. 494	p. 341	p. 28	S.1	p. 261		p. 2				p. 44	p. 133		p. 1939	p. 661	
3	Occupation	p. 823	p. 494	p. 341	p. 28	S.1		p. 133	p. 1	p. 236			p. 44	p. 133		p. 1939	p. 661	p. 484
4	Gender					S.1	p. 263											
5	Experience and training	p. 825	p. 494			S.1			p. 2	p. 236								pp. 483–484
	*Relationship with participants*																	
6	Relationship established					S.1												
7	Participant knowledge of the interviewer	p. 825				S.1									p. 34			
8	Interviewer characteristics		p. 494			S.1		p. 133										
	Domain 2: Study design																	
	*Theoretical framework*																	
9	Methodological orientation and theory	p. 826	p. 497	p. 342	p. 24	p. 105	p. 263	p. 134	p. 2	p. 237	pp. 213	p. 376	p. 38	p. 134	p. 33	p. 1941	p. 663	
	*Participant selection*																	
10	Sampling	p. 825	p. 496	p. 342	pp. 24–25	p. 105	p. 262	p. 134	p. 2	p. 236	pp. 212–213	p. 376	p. 38	p. 134	p. 33	p. 1941	p. 663	p. 484
11	Method of approach	p. 825	p. 496	p. 342	pp. 24–25	p. 105	p. 262		p. 2	p. 236	p. 213	p. 376	p. 38	p. 134	p. 33	p. 1941		
12	Sample size	p. 825	p. 496	p. 342	p. 24	p. 104	p. 263	p. 134	p. 2	p. 236	p. 213	p. 376	p. 38	p. 134	p. 34	p. 1941	p. 663	p. 483
13	Non-participation	p. 825				p. 104								p. 134				
	*Setting*																	
14	Setting of data collection	p. 825	p. 496	p. 342	p. 25	p. 105			p. 2	p. 237	p. 213	p. 376		p. 135	p. 33	p. 1942	p. 663	p. 485
15	Presence of non-participants		p. 496															
16	Description of sample	p. 827	p. 496	p. 343	p. 24	pp. 104–105	p. 263	p. 134	pp. 2–3	p. 237		p. 376	p. 38	p. 134	p. 34	p. 1941	p. 665	p. 483
	*Data collection*																	
17	Interview guide	p. 825–826	p. 496		p. 25	S.1		p. 134	p. 3	p. 237	p. 213			pp. 135–136	p. 33	p. 1942	pp. 673–675	p. 485
18	Repeat interviews					S.1												p. 484
19	Audio/visual recording	p. 826	p. 497		p. 25	p. 105	p. 263		p. 3	p. 237	p. 213	p. 376	p. 38	p. 135	p. 33	p. 1939	p. 663	p. 485
20	Field notes	p. 825	p. 497			S.1	p. 263					p. 376			p. 34			p. 485
21	Duration	p. 826	p. 496		p. 25	p. 105	p. 263		p. 2	p. 237				p. 135	p. 34	p. 1941		
22	Data saturation					p. 109	p. 263					p. 376	p. 38		p. 33	p. 1941		
23	Transcripts returned					S.1		p. 134										
	Domain 3: Analysis and findings																	
	*Data analysis*																	
24	Number of data coders		p. 497	p. 342	p. 25	S.1	p. 263		p. 3	p. 237	p. 213	p. 376	p. 38			p. 1947	p. 664	
25	Description of the coding tree	p. 826	p. 501	pp. 344–345	p. 25–27	p. 107	pp. 263–264			pp. 237–238	p. 213	p. 377	p. 40	p. 135–136	p. 35	p. 1943	pp. 664–665	
26	Derivation of themes	p. 826	p. 497	p. 342	p. 25	p. 106	p. 263	p. 134	p. 3	p. 237	p. 213	p. 376	p. 38	p. 135	p. 34	p. 1942	p. 663	p. 485
27	Software						p. 263		p. 3					p. 135		p. 1942	p. 663	
28	Participant checking				p. 25	S.1		p. 134		p. 237	p. 213		p. 38					
	*Reporting*																	
29	Quotations presented	p. 827–829	pp. 497–500	pp. 344–346	p. 26–27	p. 107	pp. 264–266	pp. 134–137	pp. 3–5	pp. 238–239	pp. 213–215	pp. 377–379	pp. 38–41	pp. 136–137	pp. 34–37	pp. 1944–46	pp. 665–669	pp. 492–494
30	Data and findings consistent	p. 829–830	pp. 500–503	p. 346	p. 27–28	pp. 109–110	pp. 266–268	pp. 137	pp. 5–6	pp. 239–p240	pp. 213–216	pp. 380–381	pp. 41–43	pp. 138–139	pp. 37–39	pp. 1946–47	pp. 669–671	pp. 494–498
31	Clarity of major themes	p. 826–829	pp. 497–500	p. 344–346	pp. 25–27	pp. 105–109	pp. 263–266	pp. 134–137	pp. 3–5	p. 238–239	pp. 213	pp. 377–380	pp. 38–41	pp. 136–137	pp. 34–37	pp. 1942–46	pp. 664–669	pp. 487–494
32	Clarity of minor themes		p. 500			pp. 105–109	pp. 263–266		pp. 3–5		p. 213	pp. 377–380		pp. 136–137		pp. 1942–46	pp. 664–669	
	Scoring																	
	Domain 1: Research team and reflexivity	5/8 (63%)	5/8 (63%)	2/8 (25%)	3/8 (38%)	8/8 (100%)	3/8 (38%)	3/8 (38%)	4/8 (50%)	2/8 (25%)	0/8 (0%)	1/8 (13%)	3/8 (38%)	3/8 (38%)	1/8 (13%)	3/8 (38%)	3/8 (38%)	3/8 (38%)
	Domain 2: Study design	11/15 (73%)	11/15 (73%)	6/15 (40%)	9/15 (60%)	14/15 (93%)	9/15 (60%)	6/15 (40%)	9/15 (60%)	9/15 (60%)	7/15 (47%)	9/15 (60%)	7/15 (47%)	10/15 (67%)	11/15 (73%)	10/15 (67%)	7/15 (47%)	8/15 (53%)
	Domain 3: Analysis and findings	5/9 (56%)	7/9 (78%)	6/9 (67%)	7/9 (78%)	8/9 (89%)	8/9 (89%)	5/9 (56%)	7/9 (78%)	7/9 (78%)	8/9 (89%)	7/9 (78%)	7/9 (78%)	7/9 (78%)	5/9 (56%)	8/9 (89%)	8/9 (89%)	4/9 (44%)
	Total	21/32 (66%)	23/32 (72%)	14/32 (44%)	19/32 (59%)	30/32 (94%)	20/32 (63%)	14/32 (44%)	20/32 (63%)	18/32 (56%)	15/32 (47%)	17/32 (53%)	17/32 (53%)	20/32 (63%)	17/32 (53%)	21/32 (66%)	18/32 (56%)	15/32 (47%)

^1^ Consolidated Criteria for Reporting Qualitative Research (COREQ) [18]. ^2^ In this table, “p.” refers to page numbers.
